# Constrained DFT-based magnetic machine-learning potentials for magnetic alloys: a case study of Fe–Al

**DOI:** 10.1038/s41598-023-46951-x

**Published:** 2023-11-13

**Authors:** Alexey S. Kotykhov, Konstantin Gubaev, Max Hodapp, Christian Tantardini, Alexander V. Shapeev, Ivan S. Novikov

**Affiliations:** 1 Skolkovo Institute of Science and Technology, Skolkovo Innovation Center, Bolshoy Boulevard 30, Moscow, 143026 Russian Federation; 2 Moscow Institute of Physics and Technology, 9 Institutskiy per., Dolgoprudny, Moscow Region 141701 Russian Federation; 3 University of Stuttgart, Postfach 10 60 37, 70049 Stuttgart, Germany; 4 Materials Center Leoben Forschung GmbH (MCL), Leoben, Austria; 5 Hylleraas Center, Department of Chemistry, UiT The Arctic University of Norway, Langnes, PO Box 6050, 9037 Tromsø, Norway; 6 Department of Materials Science, Rice University, Houston, TX 77005 USA; 7 Institute of Solid State Chemistry and Mechanochemistry SB RAS, ul. Kutateladze 18, Novosibirsk, 630128 Russian Federation

**Keywords:** Magnetic properties and materials, Computational methods, Magnetic properties and materials

## Abstract

We propose a machine-learning interatomic potential for multi-component magnetic materials. In this potential we consider magnetic moments as degrees of freedom (features) along with atomic positions, atomic types, and lattice vectors. We create a training set with constrained DFT (cDFT) that allows us to calculate energies of configurations with non-equilibrium (excited) magnetic moments and, thus, it is possible to construct the training set in a wide configuration space with great variety of non-equilibrium atomic positions, magnetic moments, and lattice vectors. Such a training set makes possible to fit reliable potentials that will allow us to predict properties of configurations in the excited states (including the ones with non-equilibrium magnetic moments). We verify the trained potentials on the system of bcc Fe–Al with different concentrations of Al and Fe and different ways Al and Fe atoms occupy the supercell sites. Here, we show that the formation energies, the equilibrium lattice parameters, and the total magnetic moments of the unit cell for different Fe–Al structures calculated with machine-learning potentials are in good correspondence with the ones obtained with DFT. We also demonstrate that the theoretical calculations conducted in this study qualitatively reproduce the experimentally-observed anomalous volume-composition dependence in the Fe–Al system.

## Introduction

Magnetism is important to be explicitly taken into account for the successful computational prediction of many properties in single-component metals^1–6^ and multi-component alloys^7–15^. In particular, magnetic properties of the constituting elements of alloys affect the phase stability^7–10^. Furthermore, magnetism can be responsible for the unusual properties like negative thermal expansion^11,12^, anomalous volume-composition dependence^13^, and the so-called “half-metallic behavior” in perovskites^14^ or full-Heusler alloys^15^. In such multi-component alloys not only magnetism is a more complex *physical* phenomenon as compared to the single-component materials, the presence of magnetism in multi-component alloys also leads to extra difficulties for its *computational* studies. Steel, a workhorse of heavy industry, is an example of such a material; it has immense variety of applications and corresponding sub-types, possessing magnetic properties due to Fe being presented in their content. Steels, as well as other Fe-based alloys, can be characterized by different magnetic orders for which the electronic ground state can be substantially different. One of the most widely used methods for simulations of ground state properties of condensed matter is density functional theory (DFT). In magnetism, however, we are are often interested in excited state properties (e.g., excited magnetic moments), while, strictly speaking, DFT is a theory for the electronic ground state: the fundamental theorem of DFT relies on a minimization of the energy in the functional space of many-body electronic wavefunction, which is the Slater determinant that avoid the electronic localization due to the spreading of all electrons on the entire orbitals (i.e., Kohn–Sham equations).

To overcome this problem constraints are introduced in the Kohn–Sham equations, either as soft constraints contributing a penalty function to the total energy^16–18^ or, as recently proposed, hard constraints solved self-consistently using the Lagrangian multiplier method^19^.

Despite the success of different flavours of DFT calculations in investigating different materials during the last 30 years they are still computationally expensive even when used them on modern supercomputers. Moreover, in computational studies of magnetic materials, including magnetic moments as an additional degree of freedom in the calculation of DFT energy, the computational time increases drastically. To overcome the limitations of DFT in size and time, requires alternative approaches. Effective interaction model (EIM) was proposed in Refs.^20,21^ and applied to the Fe-Ni system. One approach that has gained massive interest in the computational materials community over the last 15 years are machine-learning interatomic potentials (MLIPs)^22–32^. The main idea behind MLIPs is their ability to avoid running the full DFT simulation by interpolating between a relatively small training set of carefully selected single-point DFT calculations. A MLIP that has been trained on configurations that are representative for the entire configurational space appearing in a simulation then approximates local DFT energies and forces with, in principle, arbitrary accuracy—contrary to (semi-)empirical interatomic potentials. The cited MLIPs (see e.g.^22–32^) have been developed for non- or ferromagnetic materials, which do not require taking into account magnetic degrees of freedom explicitly in the functional of MLIPs. In order to approximate DFT energies that strongly depend on the magnitude and direction of the magnetic field we must enrich the functional form of MLIPs with magnetic moment features which has been proposed in a number of works^33–38^. The open problem, however, is the generation of suitable training sets. We emphasize that in addition to the configurations with non-equilibrium atomic positions and lattice parameters we also need configurations with non-equilibrium magnetic moments for the proper fitting of magnetic MLIP. We discuss the creation of such a training set in the present work.

In this paper we generalize a single-component magnetic Moment Tensor Potential^35^ (one of the recently developed MLIPs with magnetic degrees of freedom, mMTP) to the case of multi-component magnetic materials. Here, we use the  recently developed cDFT approach^19^ to evaluate the magnetism of Fe-Al system by first-principles calculations being such approach seen to efficiently describe magnetism in considering a potential-based formulation of the self-consistency problem^19^. These cDFT data will be subsequently used to generate a training set for fitting an mMTP, i.e. in addition to the configurations with equilibrium magnetic moments we also include the ones with non-equilibrium (or, perturbed) magnetic moments in the training set. An mMTP fitted to such a training set allows for equilibrating magnetic moments of a configuration starting from the perturbed ones. In other words, we consider magnetic moments on the same grounds as atomic positions: we generate a training set with both relaxed and perturbed atomic positions, fit a potential, and, finally, relax both (magnetic and “positional”) degrees of freedom of the structures with the trained potential.

We test our multi-component mMTPs on the system of Fe-Al with different concentrations and positions of Al and Fe. We demonstrate that the formation energies, the equilibrium lattice parameters, and the total magnetic moments of the unit cell predicted with the fitted mMTPs for various compounds of the Fe-Al alloy are in a good correspondence with the DFT ones. We also demonstrate that the theoretical calculations conducted in this paper reproduce the anomalous volume-composition dependence in the Fe-Al system shown in Ref.^13^ and experimentally observed^39^.

## Results and discussion

### Training set

The training set for magnetic Moment Tensor Potential (mMTP) fitting consists of different configurations of bcc Fe-Al in which the concentration of Al varies from 0 to $50\%$. We additionally include pure bcc-Al in the training set. The configurations in the training set consist of 16 atoms ($2\times 2\times 2$ conventional bcc cell). There are altogether 2012 configurations in the training set constructed as detailed below.

**Figure 1:**
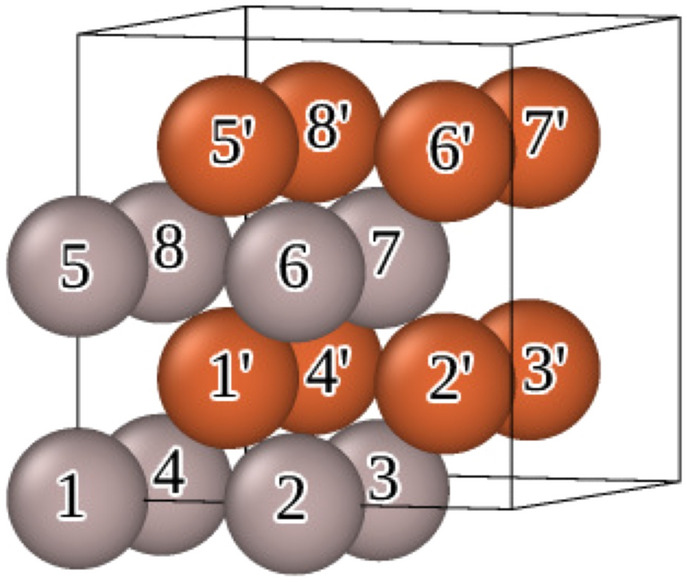
The $2\times 2\times 2$ 16-atomic supercell with the notations of atomic sites in Fe–Al structures.

**Table 1:** Construction of the training set.

Structure	Step 1	Step 2	Step 3.1	Step 3.2
Bcc-Al	3	15	75	14
Bcc-Fe	3	14	60	14
Fe$_{15}$Al$_{1}-$1	3	15	75	14
Fe$_{14}$Al$_{2}-$11’	7	15	15	14
Fe$_{14}$Al$_{2}-$15	7	14	69	14
Fe$_{14}$Al$_{2}-$13	5	14	69	14
Fe$_{14}$Al$_{2}-$17	3	15	75	14
Fe$_{13}$Al$_{3}-$124	12	14	67	14
Fe$_{13}$Al$_{3}-$157	11	14	56	14
Fe$_{13}$Al$_{3}-$368	7	15	75	14
Fe$_{12}$Al$_{4}-$1245	5	11	55	14
Fe$_{12}$Al$_{4}-$1247	11	12	58	14
Fe$_{12}$Al$_{4}-$1357	10	15	45	14
Fe$_{12}$Al$_{4}-$1368	3	8	39	14
Fe$_{11}$Al$_{5}-$13681’	5	6	30	14
Fe$_{11}$Al$_{5}-$12457	5	15	75	14
Fe$_{10}$Al$_{6}-$124568	0	0	0	0
Fe$_{10}$Al$_{6}-$123468	0	0	0	0
Fe$_{10}$Al$_{6}-$12341’5’	18	15	72	14
Fe$_{10}$Al$_{6}-$124678	4	15	74	14
Fe$_{9}$Al$_{7}-$1234568	6	15	75	14
Fe$_{8}$Al$_{8}-$12345678	3	14	70	14
Fe$_{8}$Al$_{8}-$13682’4’5’7’	3	15	69	14
Total:	134	286	1298	294

We first conduct full geometrical optimization (relaxation) of structures and get fully equilibrium structures and the ones from the relaxation path (Step 1). Next we displace atomic positions and lattice vectors and run DFT calculations with the optimization of magnetic moments (Step 2). Finally, we perturb magnetic moments in the configurations obtained after Steps 1 and 2 and get the configurations with non-equilibrium magnetic moments (Steps 3.1 and 3.2). The numbers of converged configurations for each step are given in the table. The total number of converged configurations is 2012.

Number of configurations in the training set: 2012.

For constructing the training set we start from the 23 “parent” configurations taken from Ref.^13^. These configurations differ the way Al occupies the supercell sites as given in Table 1. The notations for the supercell sites (as used in the first column of Table 1 where they denote the locations of the Al atoms) are shown in Figure 1.

For generating the configurations for the training set we first conduct full geometrical optimization (relaxation) of the structures (i.e., we minimize energies with respect to atomic positions, lattice vectors, and magnetic moments) from the left column in Table 1. We took the Fe magnetic moments of 3 $\mu _B$ and the Al magnetic moments of 0 $\mu _B$ as an initial guess. After the minimization we added the configurations from the relaxation path to the training set (see Step 1 in Table 1). After that we compress and extend lattice vectors of equilibrium configurations by 1 $\%$. Next, we take each of these configurations, and we apply random displacements to each coordinate of each atomic position in the range from $-0.1$ to 0.1 Å; we do it five times and obtain five configurations from each equilibrium, compressed and extended configuration. For the resulting displaced configurations we optimize magnetic moments (see Step 2 in Table 1). In Step 3, as opposed to Steps 1 and 2 in which we conduct DFT calculations without any constraints, we impose hard constraints on magnetic moments to obtain the configurations with non-equilibrium magnetic moments. In Step 3.1 we take each configuration from the previous step and five times randomly perturb each equilibrium atomic magnetic moment by increasing or decreasing this value by at most $15\%$, and calculate these configurations with cDFT (see Step 3.1 in Table 1). As a result, the maximum deviation from the equilibrium magnetic moment for the Fe atom could reach 0.4 $\mu _B$, for the Al atom the maximum deviation could be 0.01 $\mu _B$ (in the configurations with both Fe and Al atom). In the case of pure Al we randomly perturb magnetic moments by the values from the range $(-0.03,0.03)$ $\mu _B$. Finally, we randomly perturb equilibrium magnetic moments in the configurations obtained in Step 1 by increasing or decreasing their value by at most $50\%$ and also conduct cDFT calculations for the configurations with non-equilibrium magnetic moments, but with the equilibrium positions and lattice vectors (see Step 3.2 in Table 1). As a result, the maximum deviation from the equilibrium magnetic moment for the Fe atom could reach 1.3 $\mu _B$, for the Al atom the maximum deviation could be 0.04 $\mu _B$ (in the configurations with both Fe and Al atom). In the configurations with pure Al we randomly perturb magnetic moments by the values from the range $(-0.1,0.1)$ $\mu _B$. Thus, we obtain the training set including fully equilibrium configurations and the ones with perturbed atomic positions, lattice vectors, and magnetic moments. Number of configurations converged for each step and each structure is given in Table 1. The training set contains the total of 2012 configurations. We note that around 80 $\%$ of Fe-Al configurations converged during the DFT (cDFT) calculations with the ABINIT code. We also emphasize that all the cDFT calculations with perturbed magnetic moments and equilibrium atomic positions and lattice parameters (at Step 3.2) were converged.

For the verification of the fitted mMTPs we also generate a “verification” set. We start with the configurations obtained in Step 1 of constructing the training set, and perform Steps 2 and 3.2 (i.e., we perturb atomic positions and lattice parameters and optimize magnetic moments (Step 2) and we perturb magnetic moments at equilibrium lattice parameters and atomic positions (Step 3.2)). We thus obtain additional 336 configurations for the verification of the fitted mMTPs. Unlike for the training set, we omitted Step 3.1 for the purpose of generating the verification set (and we therefore do not call it “validation set”), because we are mostly concerned about the accuracy on equilibrium configurations.

### Fitting and verification of the ensemble of mMTPs

The results of fitting and verification of the ensemble including five mMTPs are given in Table 2. It could be seen that the uncertainty in energy, force, and stress errors is small compared to the magnitude of the errors, and the fitting errors and the errors on the “verification” set are reasonable and typical for the periodic crystal systems. We use the fitted ensemble of five mMTPs for further computations of the values of interest because from Table 2 we see no overfitting of the ensemble of five mMTPs. However, it should be noted that the force error on the “verification” set is smaller than the fitting force error and the stress error on the “verification” set is two times larger than the fitting stress error. Both the difference between force and stress errors are beyond the 95 % confidence interval. The reason may be that we constructed the “verification” set without Step 3.1 as opposed to the training set. In order to check it out we carry out an additional fivefold cross-validation.

**Table 2:** Fitting root-mean-square errors (RMSEs) and RMSEs obtained on the “verification” set for the ensemble of five mMTPs and uncertainty of the errors estimation (we provide it with the 95 % confidence interval, i.e., 2-$\sigma$ interval).

	Energy error (meV/atom)	Force error (meV/Å)	Stress error (GPa)
Fitting	3.65 ± 0.13	60 ± 3	0.42 ± 0.06
Verification	4.03 ± 0.05	48 ± 4	0.79 ± 0.10

The obtained errors are reasonable and typical for the periodic crystal systems. The ensemble of fitted mMTPs was not overfitted.

### Fivefold cross-validation

For the fivefold cross validation we combine the training and “verification” sets and create the total set of 2328 configurations. Next we split this set into five non-overlapping parts and carry out cross-validation, i.e. we fit mMTPs on four parts and validate it on the fifth part. The results are given in Table 3. From the table we conclude that the fitting and validation errors are close to each other and are within 99% confidence interval, i.e., the training set was constructed correctly.

**Table 3:** Fivefold cross-validation of mMTPs. We provide uncertainty of the errors estimation within the 99% confidence interval, i.e., 3-$\sigma$ interval.

	Energy error (meV/atom)	Force error (meV/Å)	Stress error (GPa)
Fitting	2.8 ± 0.7	57 ± 4	0.36 ± 0.06
Validation	3.1 ± 1.7	51 ± 4	0.33 ± 0.06

Fitting and validation errors are close to each other and are within 99% confidence interval.

### Formation energy, lattice parameter, and total magnetic moment for different Fe–Al compounds

For testing the predictive power of the ensemble of fitted mMTPs we compare the formation energies, the equilibrium lattice parameters, and the total magnetic moments of the unit cell predicted with the mMTPs and DFT (implemented in ABINIT) for different compounds of Fe–Al. Uncertainty of the errors estimation in all the mentioned quantities in figures and tables are provided with the 95 % confidence interval (i.e., 2-$\sigma$ interval).

**Figure 2:**
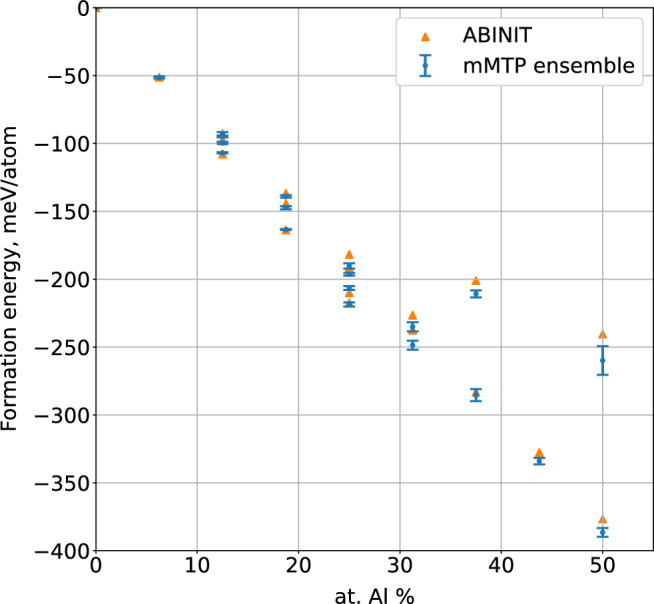
Formation energies for different compounds of Fe–Al predicted with the ensemble of mMTPs and DFT. The trend of formation energy was correctly reproduced with the ensemble of mMTPs, maximum error in formation energy prediction is 20 meV/atom for the Fe$_{8}$Al$_{8}-$13682’4’5’7’ structure.

Formation energy is given in Fig. 2. The ensemble of mMTPs correctly reproduces the formation energy trend calculated with DFT: it decreases when the concentration of Al increases. The maximum error of the formation energy prediction is around 20 meV/atom for the Fe$_{8}$Al$_{8}-$13682’4’5’7’ structure. From Fig. 2 we find the configurations with minimum energy for a given concentration of Al, i.e., closest to the convex hull: pure bcc-Fe, Fe$_{15}$Al$_1$-1, Fe$_{14}$Al$_2$-13, Fe$_{13}$Al$_3$-368, Fe$_{12}$Al$_4$-1368, Fe$_{11}$Al$_5$-12457, Fe$_{10}$Al$_6$-124678, Fe$_9$Al$_7$-1234568, and Fe$_8$Al$_8$-12345678. We provide further results for these configurations below.

**Figure 3:**
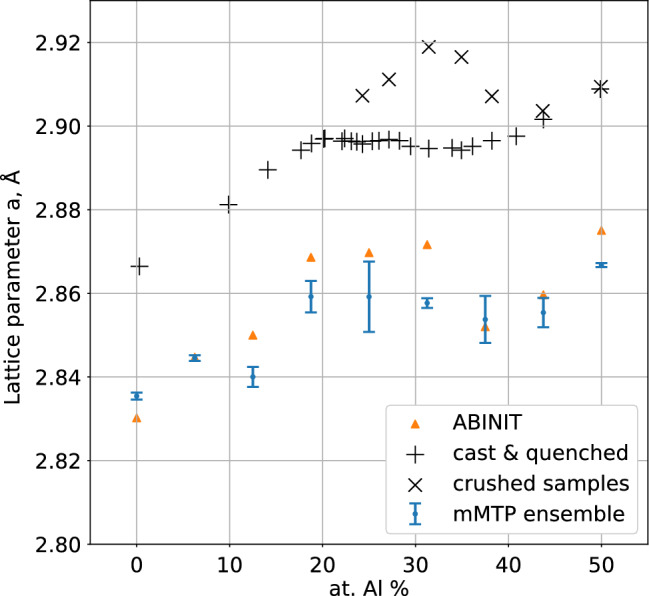
Lattice parameter dependencies on concentration of Al in the Fe–Al compounds computed with mMTP and DFT, and obtained experimentally (the experimental data are taken from Ref.^39^). The theoretical (mMTP and DFT) lattice parameters are close to each other: the maximum error in their prediction is 0.014 Å. Both the theoretical and the experimental dependencies of lattice parameters on Al concentration are anomalous: they are nonlinear at more than 18.75 % of Al concentration (at more than 31.25 % for the crushed samples).

In Fig. 3 we compare the equilibrium lattice parameters calculated with the mMTPs and DFT. As for the formation energies, the mMTP- and DFT-provided equilibrium lattice parameters are close to each other, the maximum error in their prediction is about 0.014 Å(for the Fe$_{11}$Al$_5$-12457 structure). We also observe the anomalous lattice parameter/composition dependence in the Fe–Al structures. We, first, see the nearly linear increase of the lattice parameter up to the Al concentration of 18.75 %. When the Al concentration is between 18.75 % and 31.25 % the values of lattice parameters are close to constant. Next (between 31.25 % and 37.5 %), we see the decrease in lattice parameter and, finally, another increase up to 50 %.

**Figure 4:**
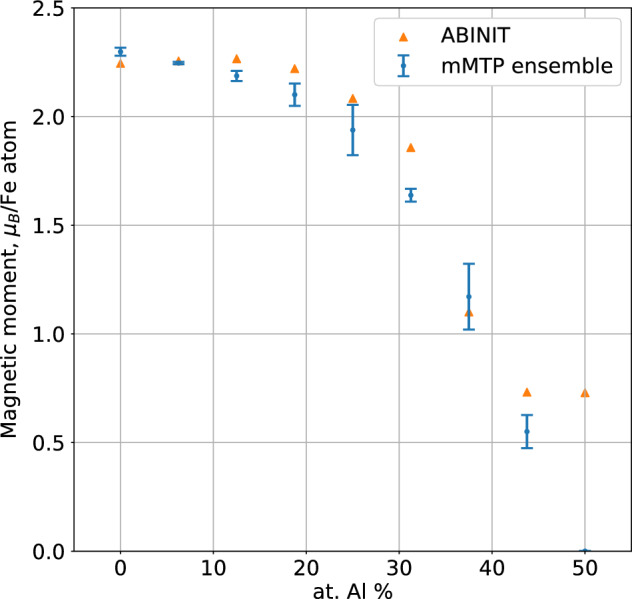
Total magnetic moment of unit cell divided by the number of Fe atoms for different compounds of Fe-Al computed with the ensemble of mMTPs and DFT. Both the mMTPs and DFT predict the decrease in total magnetic moment when the concentration of Al increases.

In Fig. 3 we also provide the experimental lattice parameters from the paper^39^. The experimental work^39^ has somewhat different lattice parameter dependencies for the differently processed samples (cast &quenched vs crushed ones), yet they both are anomalous, i.e., there is no linear dependency at more than 18.75 % Al concentration for the crushed samples (and at more than 31.25 % Al concentration for the cast &quenched ones). The origin of this anomaly itself can be successfully attributed to the change in magnetic moments of Fe atoms (see Fig. 4), as was assumed in Ref.^39^ and theoretically verified in Ref.^13^, and in this paper.

Compositional dependencies of the total magnetic moment of the 16-atomic unit cell divided by the number of Fe atoms obtained with the ensemble of mMTPs and DFT are shown in Figure 4. The overall agreement between the mMTPs and DFT is very good: we see that the total magnetic moment of the unit cell decreases when the concentration of Al increases. The values of the total magnetic moments obtained with the mMTPs and DFT are also close to each other except for the Fe$_8$Al$_8$-12345678 structure: all the fitted mMTPs in the ensemble give zero magnetic moment whereas DFT gives 0.7 $\mu _B$. Except for this discrepancy for the Fe$_8$Al$_8$-12345678 structure, from the results of this subsection we conclude that the ensemble of mMTPs fitted to the DFT data essentially reproduces the variations in formation energies, lattice parameters, and total magnetic moments calculated with DFT.

## Conclusion

In this paper we proposed the machine-learning interatomic potential with magnetic degrees of freedom (magnetic Moment Tensor Potential, mMTP) for prediction the properties of magnetic alloys. This potential was trained on data obtained with the recently developed method of cDFT calculations^19^ that allows us to compute energies of configurations with non-equilibrium (excited) magnetic moments and, thus, to consider magnetic moments as degrees of freedom along with atomic positions, atomic types, and lattice vectors. We verify the developed magnetic multi-component machine-learning potentials on the Fe-Al system. We, first, created a training set including fully equilibrium atomic positions, lattice vectors, magnetic moments and the perturbed ones for different concentrations of Fe and Al in the Fe-Al system. Next, we fitted the ensemble of five mMTPs to the cDFT data and compared the dependencies of formation energies, equilibrium lattice parameters, and total magnetic moments of unit cell on the concentration of Al atoms predicted with the ensemble of mMTPs and DFT. We concluded that the mentioned mMTP and DFT differences are minor. Both mMTPs and DFT reproduced anomalous volume-composition dependence in the Fe-Al system obtained theoretically in the previous studies and has been experimentally observed. The main difference between the mMTP and DFT results was found for the Fe$_8$Al$_8$-12345678 structure: the ensemble of mMTPs gave the local minimum with zero magnetic moments for the Fe atoms whereas DFT predicted the minimum with magnetic moments of 0.7 $\mu _B$ for the Fe atoms. Nevertheless, the rest of the results obtained with DFT and mMTP are in good correspondence.

In future, we are planning to develop an active learning algorithm for mMTP. Our confidence of developing an efficient active learning algorithm stems from the fact that with cDFT we are able to treat magnetic moments on the same footing as atomic positions; and for learning on atomic positions/geometries various successful active learning algorithms already exist. With active learning, we see in principle no obstacle in applying our methodology to predict material defect properties of other multi-component systems, e.g., of high-entropy alloys. In Ref.^40^ we have developed an MTP-based algorithm for computing stacking fault energies, surface energies, and elastic constants, for (non-magnetic) bcc random alloys and used it to predict ductility of Mo–Nb–Ta over the entire composition space. Extending it to the case of magnetic alloys should be straightforward once we have developed an active learning algorithm, and this will allow us to screen for new materials with exotic mechanical properties over a much larger space of (magnetic and non-magnetic) metallic alloys than the space that can currently be approached with todays’s state-of-the-art methods. Finally, active learning will allow us to train mMTP during molecular dynamics simulations and apply it to investigating the processes and predicting the properties of magnetic materials at finite temperature.

## Methodology

### Magnetic multi-component moment tensor potential (mMTP)

The concept of magnetic multi-component Moment Tensor Potential (mMTP) presented in the current research is based on the previously developed non-magnetic MTP for multi-component systems^41,42^ and magnetic MTP for single-component systems^35^.

The mMTP potential is local, i.e., the energy of the atomistic system is a sum of energies of individual atoms:

1 $$\begin{aligned} E = \sum _{i=1}^{N_a}E_i, \end{aligned}$$

where *i* stands for the individual atoms in an $N_a$-atom system. We note that any configuration includes lattice vectors ${{\varvec{L}}} = \{{{\varvec{l}}}_1,{{\varvec{l}}}_2,{{\varvec{l}}}_3\}$, atomic positions ${{\varvec{R}}} = \{{{\varvec{r}}}_1, \ldots , {{\varvec{r}}}_{N_a}\}$, types $Z = \{z_1,\ldots ,z_{N_{a}}\}$ (we also denote $N_{\rm types}$ by the total number of atomic types in the system), and magnetic moments $M = \{m_1,\ldots ,m_{N_a}\}$. The energy of the atom $E_i$, in turn, has the form:

2 $$\begin{aligned} E_i = \sum _{\alpha =1}^{\alpha _{\rm max}} \xi _{\alpha }B_{\alpha }({\mathfrak n}_i), \end{aligned}$$

where ${{\varvec{\xi }}} = \{\xi _{\alpha } \}$ are the “linear” parameters to be optimized and $B_\alpha$ are the so-called *basis functions*, which are contractions of the *descriptors*^25^ *of atomistic environment* ${\mathfrak n}_i$, yielding a scalar. The $\alpha _\text {max}$ parameter can be changed to provide potentials with different amount of parameters^35^.

The descriptors are composed of the radial part, i.e., the scalar function depending on the interatomic distances and atomic magnetic moments, and the angular part, which is a tensor of rank $\nu$:

3 $$\begin{aligned} M_{\mu ,\nu }({\mathfrak n}_i)=\sum _{j} f_{\mu }(| {{\varvec{r}}}_{ij}|,z_i,z_j,m_i,m_j)\underbrace{{{\varvec{r}}}_{ij}\otimes ...\otimes {{\varvec{r}}}_{ij}}_\nu \text { times }, \end{aligned}$$

where ${\mathfrak n}_i$ stands for the atomic environment, including all the atoms within the $R_\text {cut}$ distance (or less) from the central atom *i*, $\mu$ is the number of the radial function, $\nu$ is the rank of the angular part tensor, $|{{\varvec{r}}}_{ij}|$ is the distance between the atoms *i* and *j*, $z_i$ and $z_j$ are the atomic types, $m_i$ and $m_j$ are the magnetic moments of the atoms.

The radial functions are expanded in a basis of Chebyshev polynomials:

4 $$\begin{aligned} f_{\mu }(|r_{ij}|,z_i,z_j,m_i,m_j) = \sum _{\zeta =1}^{N_{\phi }} \sum _{\beta =1}^{N_{\psi }}\sum _{\gamma =1}^{N_{\psi }}c_{\mu ,z_i,z_j}^{\zeta ,\beta ,\gamma } \phi _{\zeta }(|{\varvec{r}}_{ij}|) \psi _{\beta }(m_i)\psi _{\gamma }(m_j) (R_{\rm cut} - |{\varvec{r}}_{ij}|)^2. \end{aligned}$$

Here ${{\varvec{c}}} = \{c_{\mu ,z_i,z_j}^{\zeta ,\beta ,\gamma }\}$ are the “radial” parameters to be optimized, each of the functions $\phi _{\zeta }(|{\varvec{r}}_{ij}|)$, $\psi _{\beta }(m_i)$, $\psi _{\gamma }(m_i)$ is a Chebyshev polynomial of order $\zeta$, $\beta$ and $\gamma$ correspondingly, taking values from $-1$ to 1. The function $\phi _{\zeta }(|{\varvec{r}}_{ij}|)$ yields the dependency on the distance between the atoms *i* and *j*, while the functions $\psi _{\beta }(m_i)$ and $\psi _{\gamma }(m_j)$ yield the dependency on the magnetic moments of the atoms *i* and *j*, correspondingly. The arguments of the functions $\phi _{\zeta }(|{\varvec{r}}_{ij}|)$ are on the interval $(R_{\rm min},R_{\rm cut})$, where $R_{\rm min}$ and $R_{\rm cut}$ are the minimum and maximum distance, correspondingly, between the interacting atoms. The functions $\psi _{\beta }(m_i)$ and $\psi _{\gamma }(m_j)$ are of the same structure, which we explain for the case of the former one. The argument of the function $\psi _{\beta }(m_i)$ is the magnetic moment of the atom *i*, taking the values on the $(-M_{\rm max}^{z_i},M_{\rm max}^{z_i})$ interval. The value $M_{\rm max}^{z_i}$ itself depends on the type of atom $z_i$, and is determined as the maximal absolute value of the magnetic moment for atom type $z_i$ in the training set. Similar to the conventional MTP, the term $(R_{\rm cut} - |{\varvec{r}}_{ij}|)^2$ provides smooth fading to 0 when approaching the $R_{\rm cut}$ distance, in accordance with the locality principle (1).

We note that magnetic degrees of freedom $m_i$ from (4) are collinear, i.e., they can take negative or positive values as projection onto the *Z* axis (though the choice of the axis is arbitrary). This way, in comparison to non-magnetic atomistic systems with *N* atoms, in which the amount of degrees of freedom equals 4*N* (namely 3*N* for coordinates and *N* for types), for the description of magnetic systems additional *N* degrees of freedom are introduced, standing for the magnetic moment $m_i$ of each atom. The amount of parameters entering the radial functions (Eq. 4) also increases in mMTP compared to the conventional MTP^41,42^. Namely, in MTP this number equals $N_{\mu } \cdot N_{\phi } \cdot N_{\rm types}^2$, while in mMTP it is $N_{\mu } \cdot N_{\phi } \cdot N_{\rm types}^2 \cdot N_{\psi }^2$. Thus, if we take $N_{\psi } = 2$ (which is used in the current research), the amount of the parameters entering the radial functions would be four times more in mMTP then in MTP.

We denote all the mMTP parameters by ${\varvec{\theta }}= \{{\varvec{\xi }}, {\varvec{c}} \}$ and the total energy (1) of the atomic system by $E=E({{\varvec{\theta }}})=E({{\varvec{\theta }}};M)=E({{\varvec{\theta }}};{{\varvec{L}}},{{\varvec{R}}},Z,M)$.

### Magnetic symmetrization of mMTP

The tensor (Eq. (4)) includes collinear magnetic moments in its functional form. However, it is not invariant with respect to the inversion of magnetic moments, i.e., $E({{\varvec{\theta }}};M) \ne E({{\varvec{\theta }}};-M)$, while both original and spin-inverted configurations should yield the same energy due to the arbitrary orientation of the projection axis, which we further call the *magnetic symmetry*.

We use data augmentation followed by explicit symmetrization with respect to magnetic moments to train a symmetric mMTP as we discuss below. Assume we have *K* configurations in the training set with DFT energies $E_k^{\rm DFT}$, forces ${\varvec{f}}^{\rm DFT}_{i,k}$, and stresses $\sigma ^{\rm DFT}_{ab,k}$ ($a,b=1,2,3$) calculated. We find the optimal parameters $\bar{{{\varvec{\theta }}}}$ (fit mMTP) by minimizing the objective function:

5 $$\begin{aligned} &\sum _{k=1}^{K} \Biggl [ w_{\rm e} \Biggl | \frac{E_k ({\varvec{\theta }}; M) + E_{k}({\varvec{\theta }}; -M)}{2} - E_{k}^{\rm DFT}\Biggr |^2 \\&\quad + w_{\rm f} \sum _{i=1}^{N_a} \Biggl | \frac{{\varvec{f}}_{i,k}({\varvec{\theta }};M) + {\varvec{f}}_{i,k}({\varvec{\theta }};-M)}{2} - {\varvec{f}}^{\rm DFT}_{i,k}\Biggr |^2 \\&\quad +w_{\rm s} \sum _{a,b=1}^{3} \Biggl | \frac{\sigma _{ab,k}({\varvec{\theta }};M)+\sigma _{ab,k}({\varvec{\theta }};-M)}{2} -\sigma ^{\rm DFT}_{ab,k}\Biggr |^2 \Biggr ], \end{aligned}$$

where $w_{\rm e}$, $w_{\rm f}$, and $w_{\rm s}$ are non-negative weights. By minimizing (5) we find such optimal parameters $\bar{{{\varvec{\theta }}}}$ that yield $E_k (\bar{{\varvec{\theta }}}; M) \approx E_k (\bar{{\varvec{\theta }}}; -M)$, $k = 1, \ldots , K$ (the same fact takes place for the mMTP forces and stresses), i.e., we symmetrize the training set to make mMTP learn the required symmetry from the data itself—this is called *data augmentation*.

Next, we modify mMTP to make the energy used for the simulations (e.g., relaxation of configurations) to satisfy the exact symmetry:

6 $$\begin{aligned} E^{\rm symm}(\bar{{{\varvec{\theta }}}};M) = \dfrac{E(\bar{{\varvec{\theta }}};M)+E(\bar{{\varvec{\theta }}};-M)}{2}. \end{aligned}$$

That is, we substitute the mMTP energy (1) into (6) and get a functional form which satisfies the exact identity $E^{\rm symm}(\bar{{{\varvec{\theta }}}};M) = E^{\rm symm}(\bar{{{\varvec{\theta }}}};-M)$ for any configuration. We also note that $E (\bar{{\varvec{\theta }}}) \approx E^{\rm symm}(\bar{{{\varvec{\theta }}}})$.

### Constrained density functional theory calculations

We use the cDFT approach with hard constraints (i.e., Lagrange multiplier) as proposed by Gonze et al. in Ref.^19^. One way to formulate it is to first note that in a single-point DFT calculation we minimize the Kohn-Sham total energy functional $E[\rho ; {{\varvec{R}}}]$ with respect to the electronic density $\rho =\rho (r)$ (here $\rho$ combines the spin-up and spin-down electron densities), keeping the nuclei position ${{\varvec{R}}}$ fixed. In other words, we solve the following minimization problem:

$$\begin{aligned} E_{\rm DFT}({{\varvec{R}}}) = \min _\rho E[\rho ; {{\varvec{R}}}], \end{aligned}$$

and from the optimal $\rho ^* = \mathrm{arg\,min} E[\rho ; {{\varvec{R}}}]$ we can, e.g., find magnetization $m(r) = \rho ^*_+ - \rho ^*_-$, where the subscripts denote the spin-up ($+$) and spin-down (−) densities. The magnetic moment of the *i*th atom can be found by integrating *m*(*r*) over some (depending on the partitioning scheme) region around the atom:

7 $$\begin{aligned} m_i = \int _{\Omega _i} m(r) \textrm{d}r. \end{aligned}$$

Since the minimizer $\rho ^*$ depends on ${{\varvec{R}}}$, $m_i$ are also the functions of ${{\varvec{R}}}$.

According to the cDFT approach^19^, we now formulate the problem of minimizing $E[\rho ; {{\varvec{R}}}]$ in which not only ${{\varvec{R}}}$, but also $\rho$ is allowed to change only subject to constraints (7):

$$\begin{aligned} \begin{array}{rcl} E_{\rm cDFT}(\rho, {{\varvec{R}}}, M) =&{} \min _\rho &{} E[\rho ; {{\varvec{R}}}] \\ &{} \text {subject to} &{} m_i = \int _{\Omega _i} \big (\rho _{+}(r)-\rho _-(r)\big ) \textrm{d}r. \end{array} \end{aligned}$$

The algorithmic details of how this minimization problem is solved, and how the energy derivatives (forces, stresses, torques) are computed, are described in detail in Ref.^19^.

### Computational details

We used the ABINIT code^43,44^ for DFT (and cDFT recently developed and described in Ref.^19^) calculations with $6\times 6\times 6$ k-point mesh and cutoff energy of 25 Hartree (about 680 eV). We utilized the PAW PBE method with the generalized gradient approximation. We applied constraints on magnetic moments of all atoms during cDFT calculations.

We fitted an ensemble of five mMTPs with 415 parameters in order to quantify the uncertainty of mMTPs predictions. For each mMTP we took $R_{\rm min} = 2.1 ~$ Å, $R_{\rm cut} = 4.5 ~$Å, $M_{\rm max}^{\rm Al} = 0.1 ~\mu _B$, and $M_{\rm max}^{\rm Fe} = 3.0 ~\mu _B$. The weights in the objective function (5) were $w_{\rm e} = 1$, $w_{\rm f} = 0.01$ Å$^2$, and $w_{\rm s} = 0.001$.

## Data Availability

We published the training set at (https://gitlab.com/ivannovikov/datasets_for_magnetic_MTP/-/blob/main/training_set.cfg) and the “verification” set at (https://gitlab.com/ivannovikov/datasets_for_magnetic_MTP/-/blob/main/verification_set.cfg).
